# Common Mental Disorder Symptoms among Patients with Malaria Attending Primary Care in Ethiopia: A Cross-Sectional Survey

**DOI:** 10.1371/journal.pone.0108923

**Published:** 2014-09-30

**Authors:** Markos Tesfaye, Charlotte Hanlon, Fasil Tessema, Martin Prince, Atalay Alem

**Affiliations:** 1 Department of Psychiatry, College of Public Health & Medical Sciences, Jimma University, Jimma, Ethiopia; 2 Department of Psychiatry, School of Medicine, College of Health Sciences, Addis Ababa University, Addis Ababa, Ethiopia; 3 Centre for Global Mental Health, Health Services and Population Research Department, Institute of Psychiatry, King’s College London, London, United Kingdom; 4 Department of Epidemiology and Biostatistics, College of Public Health & Medical Sciences, Jimma University, Jimma, Ethiopia; Brighton and Sussex Medical School, United Kingdom

## Abstract

**Background:**

Common Mental Disorders (CMDs) are frequent among patients attending primary care. In Africa, CMDs are often misdiagnosed as physical illnesses because many of the patients complain of somatic symptoms of mental distress. We explored whether there was difference in the levels of CMD symptoms between patients with thick film confirmed and clinical cases of malaria with negative thick film in primary care.

**Methods:**

A cross-sectional comparative study was conducted on 300 adults with a clinical diagnosis of malaria in primary care centres in Jimma, Ethiopia. Patients were recruited consecutively until 100 cases of ‘malaria’ with a negative thick film and 200 cases of malaria with a positive thick film consented to participate. The 20-item Self-Reporting Questionnaire (SRQ-20) was used to measure CMD. The non-parametric Wilcoxon rank-sum test was used to explore the association between thick film result and CMD.

**Results:**

Participants had a mean age of 28.2 (S.D = 10.9) years and the majority (57.3%) were women. The prevalence of high CMD symptoms (six or more symptoms on the SRQ-20) was 24.5%. Suicidal ideation was reported by 13.8% of the participants. CMD symptoms were significantly higher in patients who had taken medication prior to visiting the primary care (p = 0.012) and in those whose symptoms had been present for seven days or more (p = 0.041). There was no statistically significant association between level of CMD symptoms and having a negative thick film result (OR 0.98; 95%CI 0.92, 1.04) or objective presence of fever (OR 1.04; 95%CI 0.93, 1.15).

**Conclusions:**

CMD symptoms among cases of malaria did not appear to be associated with a negative thick film result. The high levels of CMD symptoms, including suicidal ideation, calls for further studies to investigate the persistence and progression of these symptoms following resolution of the acute malarial episode.

## Background

In Ethiopia, malaria was a leading cause of morbidity, mortality and health service use, accounting for 17.8% of out-patient consultations, 14.1% of hospital admissions and 21.8% of deaths in 2005/06 [1]. The diagnosis of malaria relies on the demonstration of asexual forms of plasmodia parasites on microscopic examination of blood film (thick film) and/or positive result on the Rapid Diagnostic Test (RDT) (a dipstick test for malaria specific antigens) at the primary care level [2]. Although guidelines advise primary care workers to treat for malaria after confirmatory testing, studies have found that over-prescription of antimalarial medication, either despite a negative test or without an appropriate test, has been a common practice in sub-Saharan Africa [3], [4]. A recent study of the validity of presumptive diagnosis of malaria at a rural health post showed that only 40.4% of the ‘malaria cases’ diagnosed by health workers had a positive thick film [5]. In addition, in countries like Ethiopia where people have low health service utilization of only 0.33 [1], people often practice self-treatment for malaria which may lead to partial treatment [6].

Although negative thick films may arise from partially-treated malaria, it is probable that a substantial proportion of patients receive antimalarial treatment unnecessarily. Even in centres where microscopic examination facilities are available, the validity of thick film result has been questioned. A multicentre study in northwest Ethiopia found thick film examination in health facility settings to have a sensitivity of 73.6% and specificity of 79.5% compared to gold standard thick film examination by reference readers [7]. A study on paediatric inpatients with a primary diagnosis of malaria at a teaching hospital in Ethiopia found that 41.3% of patients receiving treatment for severe malaria had a negative thick film result [8]. The latter might be due to prior partial treatment but may also arise due to presumptive over-diagnosis of malaria. Similarly studies in other African countries [9], [10] and outside of sub-Saharan Africa [11] have found over-diagnosis of malaria to be common, resulting in mismanagement of patients. Indirect evidence suggests that such mismanagement contributes to worsening health and poverty [12].

A multi-national survey in primary care settings in developing countries demonstrated that the overall prevalence of psychiatric morbidity, mostly comprising depression and anxiety, was significant, ranging from 10.6% to 17.7% across centres [13]. Similarly, a high prevalence (ranging from 18% to 27%) of psychiatric morbidity has been found in primary care and general outpatient settings in Ethiopia [14]–[16]. Nonetheless, the detection of cases of CMD in primary care in developing countries is low [17], [18]. Low mental health literacy and stigma associated with mental disorder may lead patients to present their emotional distress through physical (somatic) complaints [19]. The contribution of undetected somatisation of mental distress to misdiagnosis of malaria has previously been hypothesised [20]. Symptoms of malaria such as headache, joint pain, anorexia and fatigue are well-recognised presenting symptoms of CMD in sub-Saharan Africa and have every potential to be misdiagnosed as malaria [13], [19], [21]. In Malawi, training of primary care workers resulted in significantly higher rates of diagnosis of anxiety and depression, and significantly reduced rates of diagnosis of malaria, providing indirect evidence for the misdiagnosis of CMD as malaria in such settings [18].

The non-treatment of CMD has important public health consequences such as poor health-related quality of life and greatly increased health care utilization [22]–[24]. If undetected CMD is found to be an important factor in the misdiagnosis of malaria in primary care, this will be of great significance for the treatment and control of malaria in developing countries. As CMDs can be diagnosed and effectively managed by primary care workers in developing countries, this has the potential to also improve management of mental health problems [25]. Moreover, the Ethiopian national mental health strategy has planned to train primary care workers to detect and manage mental health problems including CMDs [26]. In light of the above, it is necessary to explore whether clinically diagnosed cases of malaria with negative thick film represent CMD cases with predominant somatic symptoms who might benefit from mental health intervention.

In this study we aimed to investigate whether there was a difference in the levels of CMD between thick film confirmed and clinical cases of malaria with negative thick film at primary care centres in Jimma, southwest Ethiopia.

## Methods

### Study design

A cross-sectional comparative study design was employed.

### Setting

Jimma is one of the zones (sub-regions) in the Oromia regional state and is located in South-western Ethiopia. Jimma is known to be a malaria endemic area [27]. There are several private clinics, three primary care centres, a district hospital, and a teaching referral hospital providing health care to the people living in the city and surrounding communities estimated to be over 120, 960 residents [28]. The study was conducted from October 2008 to February 2009 at two of the primary care centres, namely, ‘Jimma’ and ‘Higher 2’ primary care centres. The primary care centres were staffed by health officers (trained to carry out many medical duties in the absence of physicians) and nurses (degree and diploma level). Both primary care centres had laboratory technicians and were equipped with facilities for microscopic blood film examination for detection of malaria. The primary care workers are expected to send all suspected cases of malaria for a thick film examination after completing their clinical assessments. At the time of data collection there were no facilities for rapid diagnostic testing.

### Sampling

All consecutive, consenting adults whom the primary care professional diagnosed as having malaria at the respective primary care centres were invited to participate in the study until approximately 100 clinical cases of malaria with negative thick film and 200 cases of malaria with positive thick film were recruited. We excluded persons: younger than 18 years, who were too ill to be interviewed, who did not understand the language of the interviews, and who had severe forms of malaria that required inpatient treatment. In addition, patients whose initial presenting complaint was ‘malaria’ but who were diagnosed by the primary care workers to have a different condition were also excluded.

### Sample size

The sample size was calculated assuming a 15% prevalence of CMD in the thick film positive group and 30% in thick film negative ‘cases of malaria’. Assuming the ratio of positive to negative films to be 2∶1 and using 80% power with the level of significance set at 0.05, we needed 97 participants in the thick film negative and 194 in the thick film positive groups (EPI Info).

### Data collection

Two nurses and one health officer responsible for the outpatient care in each of the primary care centres were trained to recruit eligible cases. The health professionals assigned a research code number to consenting eligible participants. They also recorded the axillary temperature, thick film result, and presence of any diagnosis in addition to malaria. The health professionals were provided with a digital thermometer by the research project. Participants were sent for face-to-face interviews with the research nurses on the same day. The research nurses gathered data on socio-demographic characteristics (age, sex, marital status, occupation, religion and ethnicity), socioeconomic status (level of education, monthly income, ownership of cattle, ownership of asset, e.g. radio, refrigerator, television), help-seeking behaviour, alcohol use, khat use (an evergreen shrub, Catha edulis, that contains the amphetamine-like substance cathinone), presenting symptoms, and CMD symptoms. The hard copies of the completed questionnaires have been stored at the Department of Psychiatry, Jimma University.

Symptoms of CMD were measured using the Self-Reporting Questionnaire (SRQ-20) [29]. This 20-item scale asks about depressive, anxiety and somatic symptoms that were experienced in the preceding month and generates a continuously distributed scale score indicating level of overall psychological morbidity. The SRQ-20 has been used in several previous Ethiopian studies [30]–[33]. The validity of SRQ as a continuous scale is supported by Ethiopian studies; however, a variety of optimal cut-off points have been proposed to define probable caseness for CMD [33]–[35]. In view of this, the SRQ was treated as a continuous measurement. For the multiple logistic regression analysis, a total SRQ score of 6 and above was used to define high level of CMD symptoms.

### Data analysis

Socio-demographic, socio-economic and substance use data were summarized and presented as frequencies and percentages. The non-parametric Wilcoxon rank-sum test was used to explore the association between thick film negative cases of malaria and total SRQ scores, and separately with the psychological sub-scale of SRQ. The psychological sub-scale of SRQ has been found to have very good convergent validity as total SRQ among postnatal women [36]. It has been suggested to be more useful in detecting CMDs in medical settings [36]. Since our study looked at persons with a diagnosis of malaria, the use of the psychological sub-scale of SRQ would reduce the risk of misattribution of symptoms of malaria such as headache and fatigue to CMD. Multiple logistic regression was used to adjust for potential confounding variables of the association between CMD and thick film result. For the former, each of the domains of potential confounders i.e. socio-demographic characteristics, socio-economic status, education, marital status, diagnosis of comorbid typhoid fever, age and sex, were added to the model sequentially. Finally, all of the priori potential confounders were included to give the fully adjusted model. An exploratory multivariable analysis of factors associated thick film result was conducted to identify other factors associated with being a clinical case of malaria with a negative thick film result. The association between CMD symptoms and other variables including presence objective fever was explored using Wilcoxon rank sum test.

Ethical approval was obtained from the Ethical Review Committee of Jimma University. Participants gave written informed consent before the interviews. Participants who endorsed the SRQ-20 item asking about suicidal ideation were referred for further evaluation and treatment by mental health professionals at Jimma University Specialized Hospital.

## Results

A total of 314 primary care attendees with a diagnosis of malaria who were eligible for the study were approached. Among these, 11 refused because of lack of time to complete the interview and three did not disclose their reason for refusal. The remaining 300 participants (95.5%) were included in the descriptive analyses. Thick film results were not available for seven participants.

### Socio-demographic characteristics

The mean age of participants was 28.2 (standard deviation; SD = 10.9) years. The majority (57.3%) were women. Just over half (n = 155; 53.3%) of the sample was married. Farming was the commonest occupation (n = 80; 27.5%). The majority of participants were urban dwellers (n = 165; 56.7%). About two-fifths (n = 115; 39.4%) were non-literate. Of the 270 participants who disclosed their income, half earned less than 326 Ethiopian Birr (27 US Dollars) per month (Table 1).

**Table 1:** Characteristics of malaria patients in primary care in Jimma.

*Characteristics*	*N (%)*
Age in years (n = 291)	
Under 20 years	71 (24.4)
20 to 29 years	113 (38.8)
30 to 39 years	54 (18.6)
40 years and over	53 (18.2)
Gender (n = 288)	
Female	165 (57.3)
Male	123 (42.7)
Marital status (n = 291)	
Single	106 (36.4)
Married	155 (53.3)
Divorced/Separated	13 (4.5)
Widowed	17 (5.8)
Occupation (n = 291)	
Farmer	80 (27.5)
Student	56 (19.2)
Trader	54 (18.6)
Government employee	11 (3.8)
Daily labourer	28 (9.6)
Unemployed	52 (17.9)
Other	10 (3.4)
Place of residence (n = 291)	
Urban	165 (56.7)
Rural	126 (43.3)
Educational level (n = 298)	
No formal education	117 (40.1)
First to ninth grade	143 (49.0)
Tenth grade and above	32 (11.0)
Income in Birr (n = 270)	
0 to 299	95 (35.2)
300 to 499	91 (33.7)
500 or more	84 (31.1)
Frequency of khat use (n = 291)	
Daily	58 (19.9)
1–2 times/week	88 (30.2)
1–3 times/month	11 (3.8)
Occasionally	31 (10.7)
None	103 (35.4)
Frequency of alcohol drinking (n = 291)	
Daily	3 (1.0)
1–2 times/week	20 (6.9)
1–3 times/month	5 (1.7)
Occasionally	42 (14.4)
None	221 (76.0)

### Substance Use

Three quarters of participants had used khat at least once in their lifetime and 146 (50.2%) were using khat at least weekly in the month prior to interview. Only 40.2% reported drinking alcohol at least once in their lifetime, and 7.9% were drinking alcohol at least once weekly in the previous month (Table 1).

### Common Mental Disorder (CMD) Symptoms

The total SRQ score was positively skewed with a median score of 3 (25^th^ centile 1, 75^th^ centile 5). The prevalence of high CMD symptoms (SRQ≥6) was 24.5% (n = 69). None of the participants were given a psychiatric diagnosis by the primary care workers.

**Figure 1:**
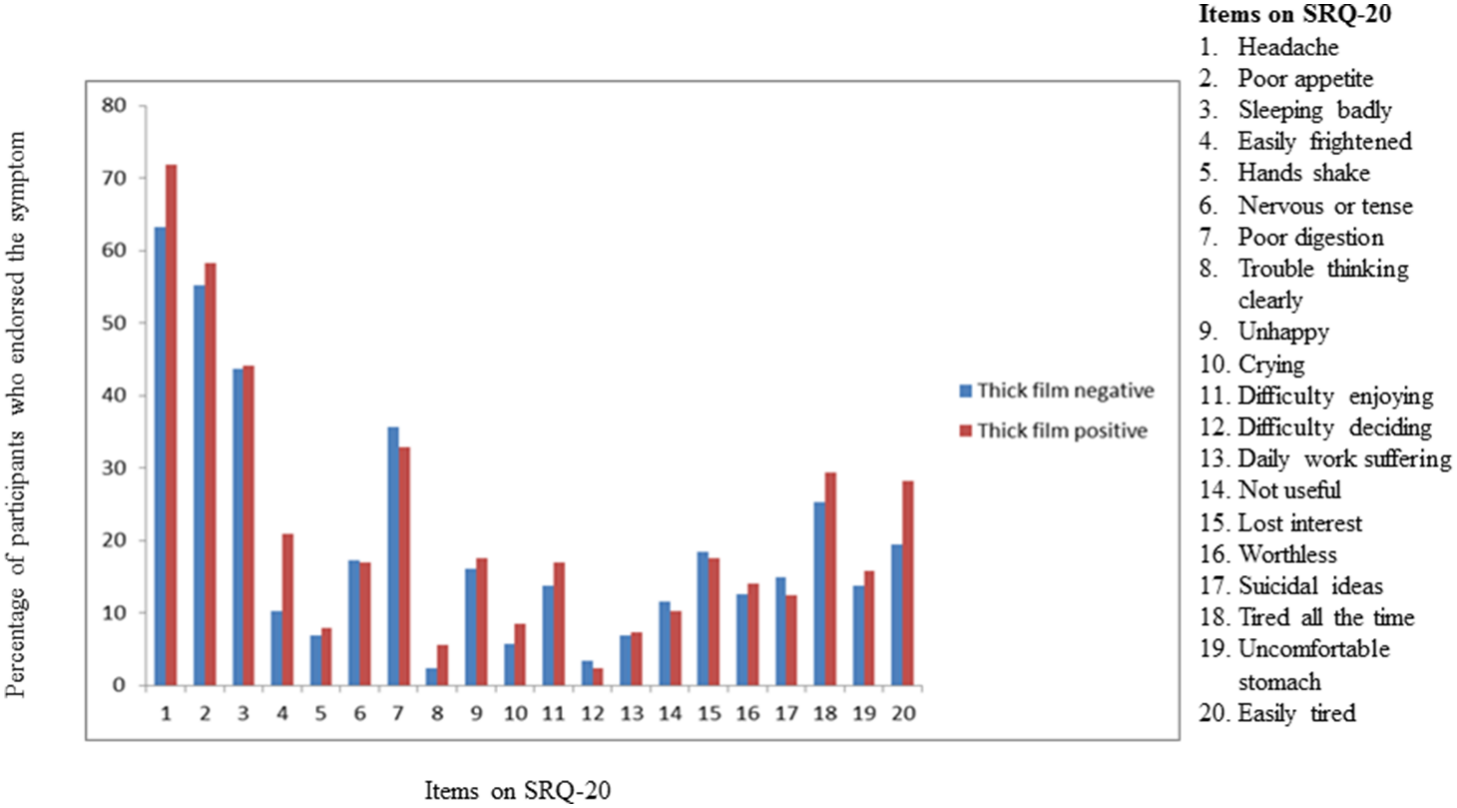
The percentage of CMD symptoms on SRQ-20 among malaria patients by thick film result in primary care in Jimma.

The most commonly reported physical symptoms on the full SRQ were: headache (68.7%), poor appetite (58.3%) and poor sleep (42.3%). The most frequent cognitive symptoms were: loss of interest in things (17.6%), feeling unhappy (17.4%), difficulty enjoying daily activities (16.6%), and feeling worthless (14.2%). Suicidal ideation was reported by 13.8% (n = 40) (Figure 1).

### Clinical and Laboratory findings

More than 80% of patients had ‘malaria-like’ symptoms for less than seven days before presenting to the primary care. One third of the patients had sought help from other places prior to presentation and a similar proportion had taken some kind of medicine.

The most frequent presenting symptoms were: headache (99.3%), fever (97.9%), chills (96.6%), and fatigue (95.9%). Of the 264 patients who had their body (axillary) temperature measured using a digital thermometer, the majority (n = 243; 92.0%) were febrile (temperature ≥37.5°Celsius) at presentation (Table 2).

**Table 2:** Clinical characteristics of malaria patients in primary care in Jimma.

*Characteristic*	*N (%)*
Blood film (n = 293)	
Negative	93 (31.7)
Falciparam malaria	70 (23.9)
Vivax malaria	128 (43.7)
Mixed falciparum/vivax	2 (0.7)
Body temperature (n = 264)	
≥37.5°C	243 (92.0)
<37.5°C	21 (8.0)
Duration of symptoms (n = 289)	
<7 days	236 (81.7)
7 days or more	53 (18.3)
Medication prior to presentation (n = 291)	
None	185 (63.3)
Analgesics/antipyretic	75 (25.8)
Chloroquine	18 (6.2)
Antibiotics	4 (1.4)
Coarthem	5 (1.7)
Unknown	4 (1.4)
Where sought help (n = 291)	
Did not seek help	184 (63.2)
Pharmacy	65 (22.3)
Health post	15 (5.0)
Private clinic	13 (4.3)
Shop	11 (3.7)
Other	3 (1.0)

Blood sample examination showed that 93 (31.7%) of the patients diagnosed as having malaria had a negative thick film result. The number of patients diagnosed with falciparum, vivax, and mixed falciparum and vivax malaria were 70 (23.9%), 128 (43.7%), and 2 (0.7%), respectively. Thirteen (5.1%) of the patients had an additional diagnosis of typhoid fever. Less than five percent of the patients reported that they suffered from chronic medical illnesses such as diabetes mellitus, hypertension or asthma.

### Bivariate and multivariable analyses

#### (i) CMD symptoms

There was no statistically significant association between a one-point increase in total SRQ score and having a negative thick film result (OR 0.98; 95%CI 0.92, 1.04) or objective presence of fever (OR 1.04; 95%CI 0.93, 1.15). Multiple logistic regression modelling found no evidence of confounding of the relationship between SRQ score and thick film result (Table 3).

**Table 3:** Association between SRQ score and having a negative thick film result among malaria patients in primary care in Jimma.

	*OR (95%CI) for total SRQ and thick film negative*
Crude odds ratio	0.98 (0.92, 1.04)
Adjusting individually for:	
Socio-economic status	0.98 (0.92, 1.05)
Educational status	0.98 (0.92, 1.05)
Age and sex	0.97 (0.91, 1.04)
Substance misuse	0.98 (0.92, 1.04)
Marital status	0.99 (0.93, 1.05)
Comorbid typhoid fever	0.98 (0.92, 1.04)
Fully adjusted model	0.97 (0.90, 1.04)
	***OR (95%CI) for SRQ psychological sub-scale and*** ***thick film negative***
Crude	0.98 (0.88, 1.08)
Adjusting individually for:	
Socio-economic status	0.99 (0.88, 1.10)
Educational status	0.98 (0.88, 1.08)
Age and sex	0.97 (0.87, 1.08)
Substance misuse	0.98 (0.88, 1.08)
Marital status	0.99 (0.88, 1.10)
Comorbid typhoid fever	0.99 (0.89, 1.10)
Fully adjusted model	0.98 (0.87, 1.10)

When analyses were repeated excluding somatic items of the SRQ, there was still no association between number of CMD symptoms and presence of a negative thick film (Table 3).

Using the two-sample Wilcoxon rank-sum test, total SRQ score was found to be significantly higher in patients who had taken medication prior to visiting the primary care (z = −2.520; p = 0.012) and in those whose symptoms had been present for seven days or more (z = −2.043; p = 0.041). There was no association between SRQ score and other variables (Table 4).

**Table 4:** Correlates of CMD symptoms among malaria patients in primary care in Jimma.

Variable	N	Z-score	P-value
Sex			
Female	159	1.485	0.14
Male	119		
Literacy			
Illiterate	108	1.668	0.10
Literate	174		
Marital status			
Married	150	1.264	0.21
Single or divorced	131		
Place of residence			
Urban	161	−1.071	0.28
Rural	120		
Thick film result			
Positive	186	0.798	0.42
Negative	89		
Objective fever			
No	26	−0.310	0.76
Yes	220		
Frequency of khat use			
Less than weekly	140	−0.987	0.32
Once a week or more	142		
Frequency of alcohol use			
Less than weekly	261	−0.408	0.68
Once a week or more	21		
Use of any medication			
No	180	−2.520	0.012
Yes	102		
Duration of symptoms			
Less than 7 days	230	−2.043	0.041
Seven days or more	49		

#### (ii) Negative blood film

Using multiple logistic regression analysis, having a negative thick film result was associated with use of chloroquine prior to presentation (OR 4.65; 95%CI: 1.69, 12.82). None of the other clinical variables were associated with a negative thick film result (Table 5).

**Table 5:** Factors associated with having negative blood film result among malaria patients in primary care in Jimma.

	*Crude OR*
Age	
Under 20	Reference
20 to 29 years	1.47 (0.77, 2.84)
30 to 39 years	1.38 (0.64, 3.00)
40 years and over	1.12 (0.50, 2.49)
Female	0.91 (0.55, 1.51)
Urban residence	1.16 (0.70, 1.92)
Non-literate	1.06 (0.64, 1.76)
No formal education	1.08 (0.65, 1.80)
Monthly income (quartiles)	
1 (lowest)	Reference
2	0.84 (0.40, 1.76)
3	1.10 (0.57, 2.16)
4 (highest)	0.82 (0.40, 1.71)
No radio	1.16 (0.66, 2.04)
No television	1.29 (0.72, 2.33)
No fridge	0.57 (0.22, 1.51)
Number of cows/oxen	
None	Reference
1 to 2	1.25 (0.64, 2.44)
3 or more	0.82 (0.43, 1.54)
Number of sheep/goats	
None	Reference
1 to 2	0.64 (0.26, 1.57)
3 or more	1.01 (0.48, 2.13)
Khat use weekly or more	1.21 (0.74, 2.00)
Alcohol weekly or more	0.97 (0.38, 2.47)
No objective fever (n = 264)	0.54 (0.17, 1.65)
Medication use pre-presentation	1.61 (0.97, 2.67)
Use of chloroquine pre-presentation	4.65 (1.69, 12.82)

## Discussion

Around one quarter of people presenting to primary care as cases of ‘malaria’ in this study from south-western Ethiopia were found to have high levels of CMD symptoms. This prevalence estimate in acutely unwell patients may have been inflated by an overlap with symptoms of malaria such as headache, loss of appetite, and fatigue. Nonetheless, cognitive symptoms of CMD that are unlikely to be symptoms of malaria such as feeling unhappy, worthlessness and expressing suicidal ideation were also high, affecting between 17.6% and 13.8% of patients. Contrary to our hypothesis, we found no difference in the number of CMD symptoms between thick film confirmed and clinical cases of malaria with a negative thick film.

The prevalence estimate for high levels of CMD symptoms in our study is higher than that found in previous studies from outpatient samples in Ethiopia. Studies from a primary care setting in south-western Ethiopia found 19% [16] whereas, another study from general hospital outpatient sample found 18% [15] to be suffering from psychiatric morbidity mainly anxiety and depression. The SRQ-20 was developed as a screening instrument rather than a diagnostic tool. The cut-off of six on the SRQ-20 is probably low for a population recruited from health facilities leading to overestimation of CMDs. Therefore, the actual prevalence of CMDs in our study population is probably lower than that suggested by our findings. On the other hand, the SRQ-20 has been found to be valid as a screening tool to detect CMD among postnatal women who have physical symptoms that could interfere with the functioning of the scale [33]. Also, unpublished data show that the SRQ-20 worked well in attendees to primary care centres in the Butajira area of Ethiopia (Charlotte Hanlon – personal communication).

None of the 40 study participants who had suicidal ideas received a diagnosis of emotional disorder by the primary care workers. Indirect evidence from sub-Saharan Africa has found that mental health training of primary care workers has improved the detection of CMDs with concomitant decrease in the proportion of patients who received a diagnosis of malaria [18]. It appears that the primary care workers in our study setting lacked the skill to detect significant emotional disorder in people presenting with malaria symptoms. Such under-recognition and under-treatment of CMDs could lead to poor quality of life, increased health service utilization and an economic burden on patients as they spend money on ineffective help-seeking [22], [23]. This finding is important in light of the plans of the Ethiopian Ministry of Health to improve access to mental health care by training primary care workers to treat priority mental disorders [26]. Training will need to focus on the typical somatic presentations of CMD in this setting, in addition to the core psychological features of depression.

The lack of association between CMD symptoms and thick film result among patients with a clinical diagnosis of malaria could be attributed to various factors. First, the limited validity of thick film result in the diagnosis of malaria in primary care could potentially have attenuated the difference (7). In particular, the apparent malaria epidemic in Jimma that coincided with our study period might have contributed as well [37]. Secondly, the relatively small number of thick film negative patients in our study may have meant that we were under-powered to detect an association. Thirdly, the high levels of symptoms of CMD among those with a thick film positive result could be attributed to the somatic symptoms of acute malaria. However, this lack of difference remained the same even when somatic items of SRQ 20 were excluded from the analysis. The cognitive CMD symptoms may result in part from a systemic immune response to acute infection [38]–[40]. This is also supported by our finding that CMDs symptoms were associated with longer duration of malaria symptoms. As a persistent systemic inflammatory response can precipitate depression in vulnerable people, people who are true cases of malaria might continue to experience CMD symptoms even after cure of their acute infection. The latter has been supported by a previous study which found persistent mixed depression-anxiety syndrome long after recovery from malaria [41]. However, the lack of a control group and the cross-sectional design of our study make it difficult to establish such a relationship. Therefore, a preferable study design to overcome this limitation would be a cohort study with follow-up mental health assessments.

The finding that high CMD symptoms were more common in those who took treatment prior to presenting to primary care services is important in the light of the widespread practice of malaria self-treatment in various settings [6]. It could be the case that those with CMD treat their ‘malaria symptoms’ inadequately. This is supported by research evidence that found poor mental health to be an important determinant of adherence to treatment for medical illnesses [20]. However, prospective studies are required to explore such an association.

Most studies on the interrelationship between malaria and mental disorders have focused on severe forms of malaria whereas there is a scanty data on the relationship in non-severe cases of malaria [42]. Our study highlights that there might be an important relationship between the latter and CMDs thus warranting large scale follow up studies. Strengths of our study include the primary care setting and the use of a comparative study design. The limited sample size and reliance on the primary care thick film result as a gold standard are limitations of our study. In addition, follow-up assessment of mental health after effective antimalarial treatment would have been more informative.

## Conclusions

A negative thick film result among clinical cases of malaria does not appear to be associated with CMD symptoms. The high levels of CMD symptoms, including suicidal ideation, among people with a diagnosis of malaria in the primary care setting in Ethiopia calls for further studies to investigate for the possibility of persistence and progression of these symptoms.
